# The emerging role of growth differentiation factor 15 as a potential disease biomarker in juvenile dermatomyositis

**DOI:** 10.1093/rheumatology/kead654

**Published:** 2023-12-07

**Authors:** Bhargavi Duvvuri, Jorge A Gonzalez-Chapa, Lauren M Pachman, Gabrielle A Morgan, Nidhi Naik, Susan Shenoi, Christian Lood

**Affiliations:** Division of Rheumatology, Department of Medicine, University of Washington, Seattle, WA, USA; Division of Rheumatology, Department of Medicine, University of Washington, Seattle, WA, USA; Division of Pediatric Rheumatology, Ann & Robert H. Lurie Children’s Hospital of Chicago, Feinberg School of Medicine, Chicago, IL, USA; Northwestern University Feinberg School of Medicine, Chicago, IL, USA; Division of Pediatric Rheumatology, Ann & Robert H. Lurie Children’s Hospital of Chicago, Feinberg School of Medicine, Chicago, IL, USA; Division of Rheumatology, Department of Pediatrics, Seattle Children’s Hospital and Research Center, University of Washington, Seattle, WA, USA; Division of Rheumatology, Department of Pediatrics, Seattle Children’s Hospital and Research Center, University of Washington, Seattle, WA, USA; Division of Rheumatology, Department of Medicine, University of Washington, Seattle, WA, USA

**Keywords:** growth differentiation factor 15, biomarker, juvenile dermatomyositis, diagnosis, prognosis

## Abstract

**Objective:**

We aimed to investigate the potential of growth differentiation factor 15 (GDF-15) as a novel biomarker for disease activity in JDM.

**Methods:**

We recruited children with juvenile myositis including JDM (*n* = 77), PM (*n* = 6) and healthy controls (*n* = 22). GDF-15 levels in plasma were measured using ELISA. Statistical analyses were performed using non-parametric tests.

**Results:**

Levels of GDF-15 were significantly elevated in JDM compared with healthy controls (*P* < 0.001). GDF-15 levels exhibited strong positive correlations with DASs, including the DAS total score, DAS skin score, DAS muscle score and Childhood Myositis Assessment Scale. Additionally, GDF-15 levels could differentiate between active disease and remission based on the Physician Global Assessment of muscle score. Positive correlations were observed between levels of GDF-15 and creatine kinase, neopterin and nailfold end row loops, indicating the potential involvement of GDF-15 in muscle damage, immune activation and vascular pathology. Receiver operating characteristics curve analysis showed GDF-15 to be more effective in assessing disease activity in JDM than creatine kinase [area under the curve (AUC) 0.77, *P* = 0.001 and AUC 0.6369, *P* = 0.0738, respectively].

**Conclusion:**

GDF-15 may serve as a valuable biomarker for assessing disease activity in JDM. It exhibits better sensitivity and specificity than creatine kinase and the levels correlate with various DASs and functional measures. GDF-15 may provide valuable information for treatment decision making and monitoring disease progression in JDM.

Rheumatology key messages

- GDF-15 levels are elevated in patients with juvenile myositis.
- GDF-15 is a novel potential biomarker for the assessment of JDM activity.
- GDF-15 is superior to creatine kinase in stratifying JDM patients based on muscle-based disease activity.

## Introduction

JDM is a rare, chronic autoimmune disorder characterized by muscle inflammation and skin rash [1]. The diagnosis and management of JDM pose significant challenges due to its heterogeneity and complex pathogenesis [2]. Thus there is an urgent need for reliable biomarkers that can aid in the early detection, assessment of disease activity and prediction of treatment response. We recently observed the involvement of mitochondria in JDM pathogenesis, with mitochondrial dysfunction being a prominent feature in muscle tissue contributing to local damage and inflammation [3]. Whether markers of mitochondrial dysfunction could be helpful in monitoring disease activity, progression and/or treatment response in JDM is not known. One emerging marker of mitochondrial dysfunction and muscle damage is growth differentiation factor 15 (GDF-15), a mitomyokine (e.g. a myokine released by muscle cells, as well as a mitokine; a marker of mitochondrial stress), suggested as a promising biomarker in several autoimmune and inflammatory diseases such as diabetes mellitus type 1, anti-phospholipid syndrome and even systemic lupus erythematosus [4–6]. Moreover, GDF-15 levels are higher in individuals with idiopathic inflammatory myopathies, including conditions such as inclusion body myositis, PM and DM [7, 8], and are associated with serious complications such as myocardial injury [8].

GDF-15, a TGF-β superfamily member, is a stress-responsive cytokine primarily secreted in response to inflammation, tissue injury or cellular stress [9]. It exerts pleiotropic effects, including regulation of apoptosis, inflammation and tissue remodelling [10]. In recent years, studies have begun to shed light on the potential involvement of GDF-15 released from muscle in response to mitochondrial dysfunction [11]. These findings suggest that GDF-15 may serve as a valuable biomarker for assessing disease activity and progression in other diseases with muscle involvement such as JDM. Of note, it has been observed that individuals with myopathies linked to mitochondrial dysfunction exhibit elevated levels of GDF-15 in their serum. Remarkably, this elevation is not observed in patients with metabolic myopathies or muscular dystrophy [12, 13].

These previous findings suggest the potential use of GDF-15 as a diagnostic marker to differentiate myopathies. In this study we explored whether GDF-15 levels could reflect disease activity in JDM. In brief, our data demonstrate that GDF-15 levels are elevated in JDM and associated with active disease, predicting disease activity even better than the currently used laboratory measure, creatine kinase (CK). Harnessing the diagnostic potential of GDF-15 could provide new insights into underlying mechanisms of JDM pathogenesis and facilitate personalized treatment strategies for enhanced patient care.

## Methods

### Participants and ethics approval

We collected banked samples from children from two centres, Children’s Hospital of Chicago and Seattle Children’s Hospital, with JDM (*n* = 57 and *n* = 20, respectively), juvenile PM (*n* = 6) and healthy controls (HCs) (*n* = 22). Blood specimens from HCs and JDM patients were obtained using age-appropriate informed written consent according to the Declaration of Helsinki, and with ethical approval from the University of Washington and Ann & Robert H. Lurie Children’s Hospital of Chicago Institutional Review Boards (2008: 13457, 2001: 11715, 2010: 14117 and 14426). Patient demographics including age, gender and race were collected as shown in Supplementary Table S1, available at *Rheumatology* online. Disease severity and activity measures were collected, including the presence of calcinosis, DAS, Childhood Myositis Assessment Scale (CMAS), Physician Global Assessment of Disease Activity (PhGA) and nailfold end row loops (NERLs) were obtained at the time of enrolment. The use of immunosuppressive medications was also recorded. The independent institutions used different disease activity measures, with the Chicago cohort using DAS and the Seattle cohort using PhGA. Myositis-specific antibodies [14] were assessed at the Oklahoma Medical Research Foundation Clinical Immunology Laboratory (Oklahoma City, OK, USA).

### Human GDF-15 DuoSet ELISA

GDF-15 levels in plasma (dilution 1:10) were measured using the human GDF-15 DuoSet ELISA (DY957, R&D Systems) following the manufacturer’s instructions.

### Statistical analyses

Statistical analyses were performed with GraphPad Prism 9.4.0 (GraphPad Software, San Diego, CA, USA). For group analyses, the Mann–Whitney *U* test was utilized, while Spearman’s correlation was used for correlation analyses. Receiver operating characteristics (ROC) curve analysis was conducted to assess the diagnostic ability of the tested variables. For post hoc multiple comparisons following a significant Kruskal–Wallis test, Dunn’s multiple comparison test was employed. *P*-values were considered significant at <0.05, <0.01 and <0.001.

## Results

### Patient population

Supplementary Table S1, available at *Rheumatology* online, lists baseline demographics, characteristics, disease severity and activity data for participants enrolled in the study. The median age of patients with JDM is 9.9 years for the Chicago cohort and 9.7 years for the Seattle cohort, while the HC cohort was slightly older, with a median age of 13.2 years. The duration of the disease varied among individuals, with median values of 38.4 months for the Chicago cohort and 37.2 months for the Seattle cohort. The majority of JDM patients were females, with 75% females in the Chicago cohort, 72% in the Seattle cohort and 55% in the HC cohort. In terms of racial distribution, the Chicago cohort had the highest percentage of White patients (73%), followed by the HC cohort (55%), and the Seattle cohort had the lowest percentage of White patients (48%). The Seattle cohort included 5% African American participants, while the Chicago cohort and the HC cohort had 9% and 23% African American participants, respectively.

The occurrence of certain autoantibodies differs between the two cohorts. In the Chicago cohort, 28% of patients tested positive for anti-p155 antibodies, 26% tested positive for anti-MJ/NXP antibodies, 7% for anti-MDA5 antibodies and 16% for anti-Mi2 antibodies. In comparison, the Seattle cohort had 27%, 18%, 0% and 16% positive results for the same antibodies, respectively.

Treatment approaches differed among the cohorts. In the Chicago cohort, 33% of patients received CSA, 31% received IVIG, 49% received MTX, 40% received steroids, 47% received MMF and 43% received HCQ. In the Seattle cohort, only 19% were receiving IVIG, with less use of most of the other drugs as well, including CSA and MMF.

### Levels of GDF-15 are associated with DASs and functional measures

**Figure 1.**
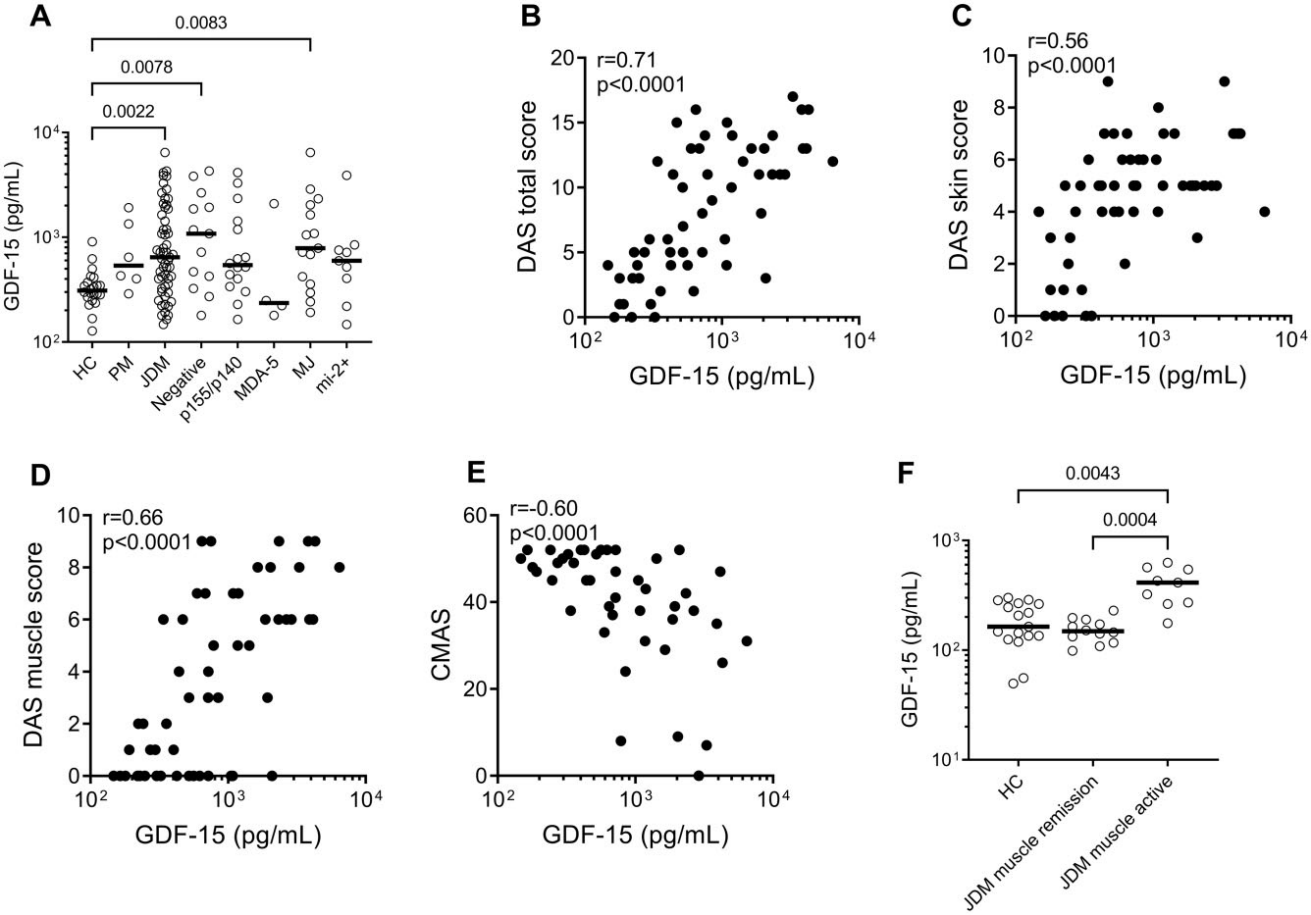
GDF-15 levels in juvenile myositis (JDM and juvenile PM) and associations with disease activity markers. (**A**) Levels of GDF-15 in plasma among different groups, including HCs (*n* = 22), PM (*n* = 6) and JDM (*n* = 57). The JDM group was further stratified based on myositis-specific antibodies: negative (*n* = 13), p155/p140 (*n* = 16), MDA5 (*n* = 4), MJ (*n* = 15) and Mi-2^+^ (*n* = 9). Correlation between GDF-15 levels and the following parameters: (**B**) DAS total score, (**C**) DAS skin score, (**D**) DAS muscle score and (**E**) CMAS in JDM patients. (**F**) Association between GDF-15 levels and the presence of active JDM disease based on the PhGA muscle score. Active disease defined was as a PhGA muscle score ≥1. Group comparisons were assessed using the Kruskal–Wallis test with Dunn’s multiple comparisons and correlations were assessed using the non-parametric Spearman correlation test. For panels A–E, the Chicago cohort was used; for panel F, the Seattle cohort was used

Levels of GDF-15 were significantly higher in JDM and PM compared with HCs (Fig. 1A). The Seattle and Chicago cohorts used different disease activity measures. Seattle used the CMAS, 8-item Manual Muscle Test (MMT8) and PhGA, whereas Chicago used DAS measures. Levels of GDF-15 exhibited a strong positive correlation with DASs, including the DAS total score (Fig. 1B), DAS skin score (Fig. 1C), DAS muscle score (Fig. 1D) and CMAS (Fig. 1E). Additionally, levels of GDF-15 demonstrated the ability to differentiate between active muscle involvement (defined as a PhGA muscle score ≥1) and remission (PhGA muscle score = 0) or HCs based on the PhGA muscle score (Fig. 1F). For disease activity correlation analyses for the Seattle cohort, see Supplementary Fig. S1, available at *Rheumatology* online. For analyses of other muscle enzymes, including aspartate aminotransferase, see Supplementary Fig. S2, available at *Rheumatology* online. These findings suggest that GDF-15 levels are associated with the overall disease activity and muscle function in JDM patients regardless of measures used clinically.

### Association of GDF-15 with other biomarkers and diagnostic utility

**Figure 2.**
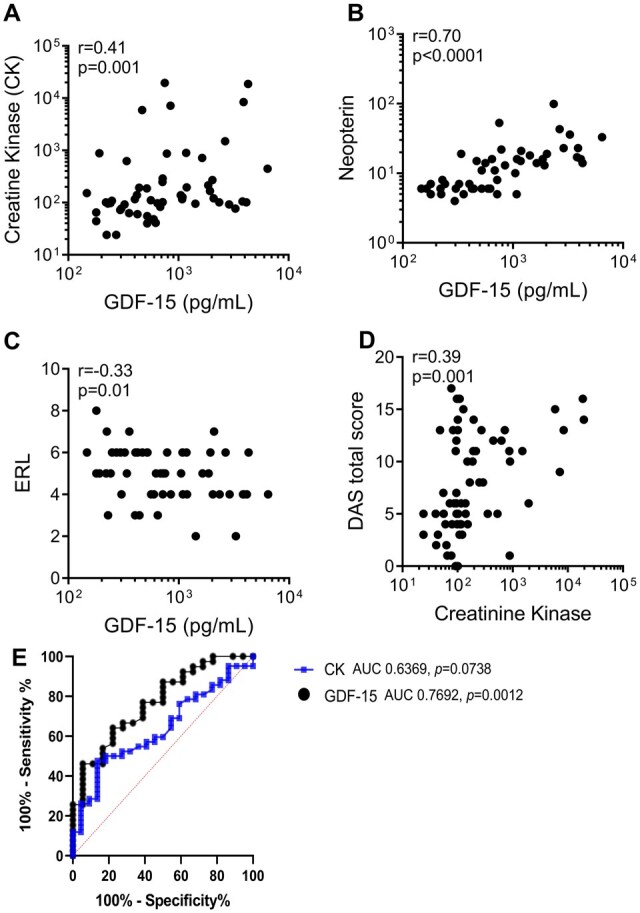
Levels of GDF-15 in JDM and associations with inflammatory markers. Correlation between GDF-15 levels and (**A**) CK, (**B**) neopterin and (**C**) end row loops (ERLs). (**D**) Correlation between CK and DAS total score using the Spearman correlation test. (**E**) ROC curve comparing CK and GDF-15 levels for distinguishing between the active or remission state based on the DAS muscle score in JDM patients. All analyses were done for the Chicago cohort

Our study revealed a positive correlation between levels of GDF-15 and another muscle-derived marker, CK (Fig. 2A). GDF-15 also showed positive correlations with neopterin (Fig. 2B) and NERLs (Fig. 2C), indicating its potential involvement in the inflammatory and vascular response. While a positive correlation was observed between CK and DAS total score (Fig. 2D), the association was comparatively weaker than that observed between DAS and GDF-15 (Fig. 1B). Through a comprehensive ROC curve assessment, GDF-15 notably outperformed CK in accurately evaluating disease activity in JDM (Fig. 2E). Specifically, the area under the curve values revealed a pronounced efficacy for GDF-15 at 0.7692 (95% CI 0.6418, 0.8966; *P* = 0.0012) compared with CK at 0.6369 (95% CI 0.4996, 0.7742; *P* = 0.0738).

Further delineation of the ROC curve analysis underscored the heightened sensitivity and specificity of GDF-15 over CK in differentiating between patients manifesting active disease and those in remission. A pivotal cut-off for GDF-15 was established at 642.4 pg/ml, reflecting a sensitivity rate of 64% and a specificity rate of 78%. Importantly, this threshold translates to a likelihood ratio of 2.88, offering a more definitive criterion for identifying active disease states. For this evaluation, the 95% CIs for sensitivity and specificity were 48.42 to 77.26% and 54.79 to 91%, respectively.

## Discussion

The identification of reliable biomarkers that reflect disease activity and functional impairment is crucial for accurate diagnosis, monitoring treatment response and predicting outcomes. In this study we investigated a novel biomarker, GDF-15, and determined associations with DASs and functional measures in JDM patients to elucidate its potential as a biomarker for disease activity and severity.

Current traditional JDM disease activity measures rely on clinical evaluation of muscle weakness with CMAS or MMT8 scales, which can be unreliable due to effort from children and other pitfalls such as the ceiling effect [15]. There are few laboratory markers, such as CK, that are available to reflect active muscle disease, and this may be falsely normal, decreased or unreliable later in the disease course when muscle atrophy or fibrosis has set in [16]. Our results demonstrate significantly higher concentrations of GDF-15 in juvenile myositis patients, including JDM and PM, as compared with HCs, consistent with what has been observed in adult IBM [7]. GDF-15 can be released by muscle cells upon cellular stress and death, as observed in exercise, sarcopenia and mitochondrial myopathy [17], also associated with regenerating and denervated muscle fibres in idiopathic inflammatory myopathies [7]. Importantly, GDF-15 levels correlated strongly with markers of disease activity and muscle function. Of note, GDF-15 could stratify patients based on disease activity, emphasizing its potential clinical utility.

The distinction between the elevation of GDF-15 and the release of CK in response to muscle damage warrants consideration. Although both markers can be released due to muscle damage, studies suggest that GDF-15 induction is specifically associated with mitochondrial dysfunction [18]. It is important to note that chronically elevated levels of GDF-15 have been implicated in contributing to muscle pathology, including the development of muscle atrophy [19]. Furthermore, we showed in our results by using an ROC curve analysis that GDF-15 had superior sensitivity and specificity in stratifying patients based on disease activity as compared with CK. GDF-15 values <642.4 pg/ml have a higher likelihood of being true negatives, while values above this threshold are more likely to be true positives. Therefore, this cut-off value can be considered a reliable indicator for JDM active disease, with a reasonable balance between sensitivity and specificity.

Although our study only allowed for associative analyses, GDF-15 may also participate in JDM pathogenesis. Supporting this hypothesis, recent work in an animal model of cancer demonstrated that neutralizing GDF-15 improved muscle function and physical performance, suggesting GDF-15 is a potential therapeutic target in myopathies [20]. Furthermore, GDF-15 has been proposed to promote mitochondrial dysfunction [18] and to regulate immune cell activation, cytokine production and fibrotic processes [21], all of which are implicated in JDM pathophysiology [2]. We consistently found that levels of GDF-15 were associated with NERL, an indicator of microvascular abnormalities, and of neopterin, a proposed marker of immune and macrophage activation [22]. These findings suggest that GDF-15 may be involved in the inflammatory response and disease progression, potentially influencing immune activation and the reported vasculopathy in JDM [23]. Our study is subject to several limitations. Primarily, we were unable to procure longitudinal samples, which restricts our capacity to comprehensively evaluate the temporal dynamics of GDF-15 marker fluctuations and its responsiveness to alterations in disease activity over time.

Moreover, our study lacks a comparative framework involving other paediatric muscle conditions and autoimmune diseases. This exclusion limits our ability to determine the specificity of our findings and poses a constraint on delineating the distinctive role and behaviour of the GDF-15 marker in JDM, as opposed to other relevant paediatric conditions.

We recognize these limitations and suggest that future research endeavours might focus on addressing these gaps to forge a more holistic understanding of the potential role of GDF-15 in JDM.

In conclusion, JDM patients have elevated levels of the mitomyokine GDF-15. Given its strong association with DASs, as well as superiority to CK, we propose that GDF-15 may be a novel and clinically useful biomarker of muscle activity in JDM. Further research with larger cohorts is warranted to validate these findings and elucidate the underlying mechanisms through which GDF-15 contributes to disease pathogenesis and progression in JDM. Additionally, investigating the utility of GDF-15 in combination with other established biomarkers and clinical assessments may enhance its diagnostic and prognostic value.

## Supplementary Material

kead654_Supplementary_Data

## Data Availability

Data are available upon reasonable request.
