# Supplementary material for: The emerging role of growth differentiation factor 15 as a potential disease biomarker in juvenile dermatomyositis
Source: Rheumatology (Oxford). 2023 Dec 7;64(2):805–9. doi: 10.1093/rheumatology/kead654 (PMC11781582; doi:10.1093/rheumatology/kead654)
Supplement: kead654_Supplementary_Data [file kead654_supplementary_data.zip › rhe-23-1457-File002.docx]

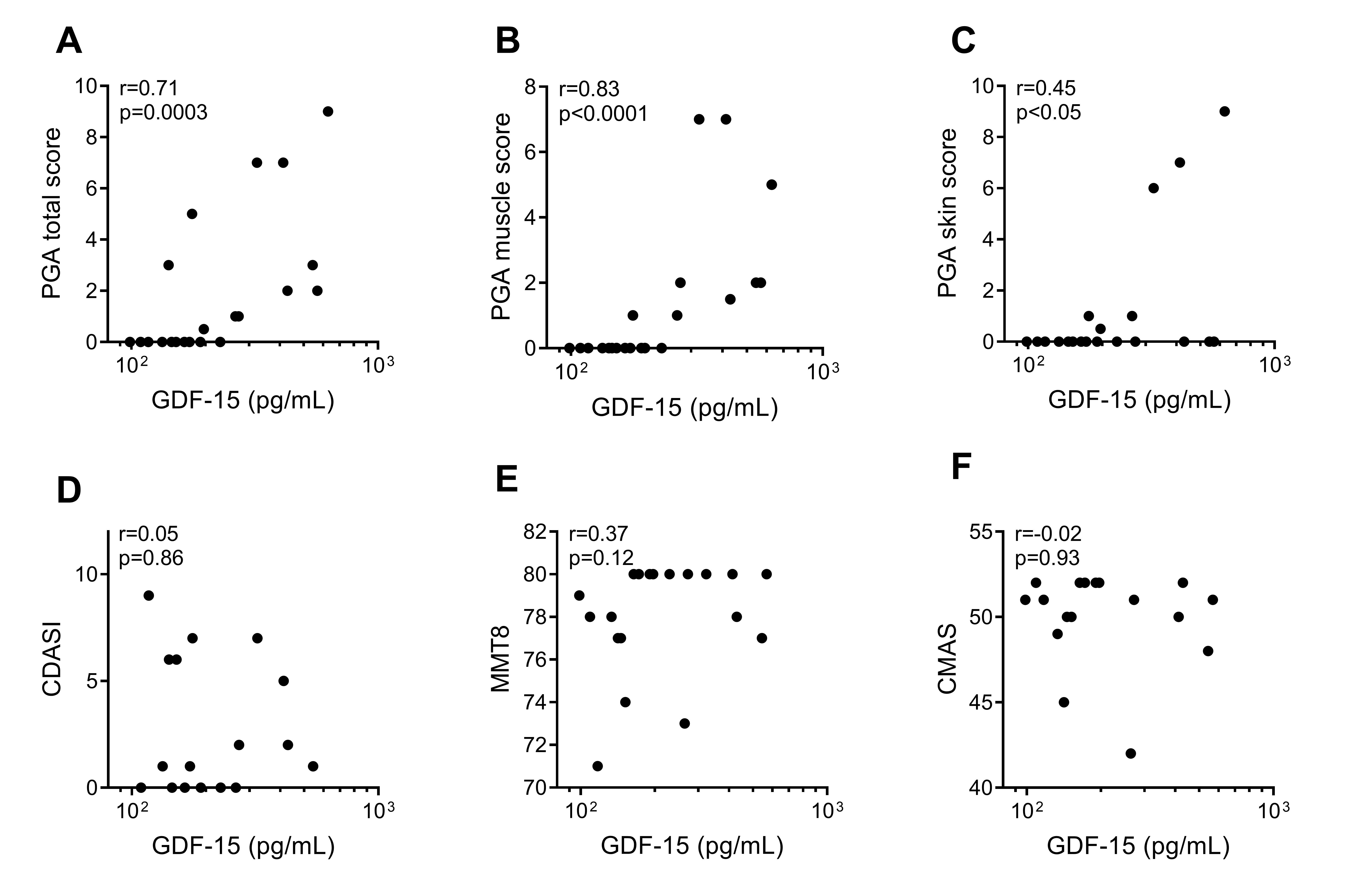


**Supplementary Figure S1. Evaluation of GDF-15 correlations with various clinical parameters in JDM.** Correlation of GDF-15 with (A) Patient Global Assessment (PGA) total score, (B) PGA muscle score, (C) PGA skin score, (D) Cutaneous Dermatomyositis Disease Area and Severity Index (CDASI) score, (E) Manual Muscle Testing using 8 muscles (MMT8), and (F) with Childhood Myositis Assessment Scale (CMAS). The correlations were assessed employing non-parametric statistical methods, specifically Spearman rank-order correlation tests.


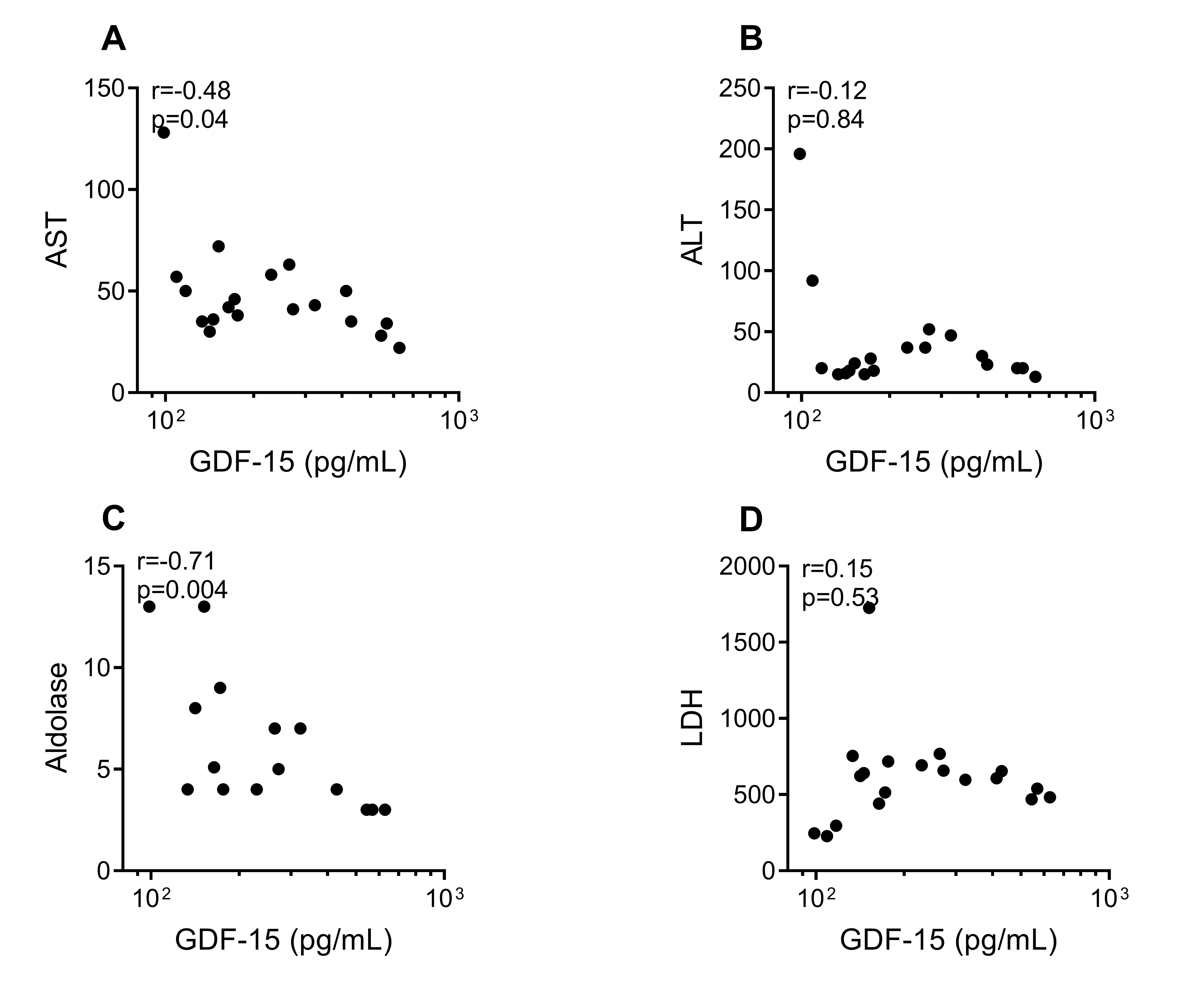


**Supplementary Figure S2. Analysis of correlations between GDF-15 and select biochemical markers in JDM.** Correlations of GDF-15 with (A) Aspartate Aminotransferase (AST), (B) Alanine Aminotransferase (ALT), (C) Aldolase, and (D) Lactate Dehydrogenase (LDH). These correlations were assessed using non-parametric statistical approaches, Spearman rank-order correlation tests were used.
