# Supplementary material for: The emerging role of growth differentiation factor 15 as a potential disease biomarker in juvenile dermatomyositis
Source: Rheumatology (Oxford). 2023 Dec 7;64(2):805–9. doi: 10.1093/rheumatology/kead654 (PMC11781582; doi:10.1093/rheumatology/kead654)
Supplement: kead654_Supplementary_Data [file kead654_supplementary_data.zip › rhe-23-1457-File003.docx]

**Supplementary Table S1**. Baseline Characteristics of Juvenile Myositis Cohort by center collected

| Cohort (N= 78) | JDM - Chicago | JDM - Seattle | HC |
| --- | --- | --- | --- |
| Number (n, #) | 57 | 21 | 22 |
| Age in years, median (range) | 9.9 (2.4-24.5) | 9.7 (4.0-19.2) | 13.2 (4.1-19.0) |
| Disease duration months, median (range) | 38.4 (0.6-186.4) | 37.2 (0-193.2) | N/A |
| Female (%) | 43/57 (75%) | 15/21 (72%) | 12/22 (55%) |
| Race - White (%) | 42/57 (73%) | 10/21 (48%) | 12/22 (55%) |
| Race – African American (%) | 5/57 (9%) | 1/21 (5%) | 5/22 (23%) |
| Race – Asian (%) | 1/57 (2%) | 1/21 (5%) | 1/22 (5%) |
| Race – Native American (%) | 0/57 (0%) | 1/21 (5%) | 0/22 (0%) |
| Ethnicity – Hispanic (%) | 9/57 (16%) | 8/21 (38%) | 4/22 (18%) |
| Calcinosis (%) | 15/57 (26%) | 1/21 (5%) | N/A |
|  |  |  |  |
| Myositis Antibodies |  |  |  |
| Anti-p155 (%) | 16/57 (28%) | 4/15 (27%) | N/A |
| Anti-MJ/NXP (%) | 15/57 (26%) | 2/11 (18%) | N/A |
| Anti-MDA5 (%) | 4/57 (7%) | 0/12 (0%) | N/A |
| Anti-Mi2 (%) | 9/57 (16%) | 3/19 (16%) | N/A |
| Anti-RNP (%) | 0/57 (0%) | 0/15 (0%) | N/A |
|  |  |  |  |
| Disease Activity, median (range) |  |  |  |
| DAS total | 7.5 (0-17) | ND | N/A |
| DAS skin | 5 (0-9) | ND | N/A |
| DAS muscle | 3 (0-9) | ND | N/A |
| PG total | ND | 0 (0-9) | N/A |
| PG muscle | ND | 0 (0-7) | N/A |
| PG skin | ND | 0 (0-9) | N/A |
| CDASI | ND | 1 (0-9)^a^ | N/A |
| CMAS | 45 (0-52)^d^ | 51 (42-52)^b^ | N/A |
| MMT8 | ND | 78 (71-80)^c^ | N/A |
| Creatinine kinase (U/L) | 114 (24-19559) | 112 (0.4-287) | N/A |
| LDH (U/L) | ND | 608 (228-1726) | N/A |
| AST (U/L) | ND | 42 (22-128) | N/A |
| ALT (U/L) | ND | 23 (13-196) | N/A |
| Aldolase (U/L) | ND | 5 (3-13) | N/A |
| Neopterin (nmol/L) | 11 (4-99)^e^ | ND | N/A |
| NERL (n) | 5 (2-8)^f^ | ND | N/A |
|  |  |  |  |
| Treatments received |  |  |  |
| CSA (%) | 19/57 (33%) | 0/21 (0%) | N/A |
| IVIG (%) | 18/57 (31%) | 4/21 (19%) | N/A |
| MTX (%) | 28/57 (49%) | 9/21 (43%) | N/A |
| Steroids (%) | 23/57 (40%) | 5/21 (24%) | N/A |
| MMF (%) | 27/57 (47%) | 2/21 (10%) | N/A |
| HCQ (%) | 25//57 (43%) | 11/21 (52%) | N/A |
| No treatment (%)^1^ | 25/57 (44%) | 6/21 (29%) | N/A |
|  |  |  |  |

^1^No treatment includes all immunosuppressive treatments, including biologicals, steroids, and HCQ.

DM: Juvenile Dermatomyositis, HC: Healthy Control, N/A: Not Available, ND: No Data

Anti-p155: Autoantibody against p155 protein, Anti-MJ/NXP: Autoantibody against MJ/NXP protein, Anti-MDA5: Autoantibody against MDA5 protein, Anti-mi2: Autoantibody against mi2 protein, Anti-RNP: Autoantibody against RNP protein, DAS: Disease Activity Score, CDASI: Cutaneous Dermatomyositis Disease Area and Severity Index, CMAS: Childhood Myositis Assessment Scale, MMT8: Manual Muscle Testing, 8 muscles, CSA: Cyclosporine, IVIG: Intravenous Immunoglobulin, MTX: Methotrexate, MMF: Mycophenolate Mofetil, HCQ: Hydroxychloroquine.

Creatinine kinase levels less than 250 U/L are within the normal range.

^a^17/21, ^b^19/21, ^c^17/21, ^d^46/57, ^e^50/57, ^f^55/57.
